# B lymphocyte mediated antigen presentation of plasmid DNA

**DOI:** 10.1186/2051-1426-1-S1-P204

**Published:** 2013-11-07

**Authors:** Viswa Teja Colluru, Douglas G McNeel

**Affiliations:** 1 Department of Medicine, University of Wisconsin-Madison, Madison, WI, USA

## Background

DNA vaccination is a safe and economical therapeutic modality that has demonstrated robust elicitation of (cellular and humoral) immunity and remarkable pre-clinical efficacy in over 30 disease models, including those of breast, prostate and colon malignancies, multiple myeloma, lymphoma and fibrosarcoma. In spite of being perched on the verge of revolutionizing the global vaccine scenario for the last 20 years, DNA vaccines have been relatively unsuccessful in several human trials so far, while achieving ‘standard of care’ status in other large animals like dogs and horses. Recent work has also shed light on the innate adjuvant effect of double-stranded DNA and its role in establishing an adaptive immune response, opening several new avenues for investigation. This prevailing scenario warrants a re-examination of the mechanism of plasmid DNA induced immunity, with a focus on relevant human cell systems.

## Methods

Cell types enriched from primary human PBMC were assayed for spontaneous plasmid DNA uptake, transgene production and antigen presentation. Plasmid DNA labeled with fluorescent peptide nucleic acid (PNA) probe was used to detect uptake of plasmid DNA after co-incubation. Transgene production after co-incubation was tested using quantitative RT-PCR and flow cytometry. Antigen presentation was examined using cells from patients with known, pre-existing T cell responses to one or more tumor antigens (PAP - prostatic acid phosphatase, SSX2 - synovial sarcoma breakpoint-2). Different antigen-presenting cell (APC) subsets were enriched and co-incubated with T lymphocytes along with either an empty vector or plasmid DNA encoding the relevant tumor antigen and assayed for expansion of T cells after 7-10 days.

## Results

Uptake of plasmid DNA was primarily exhibited by dendritic cells (CD11c+), monocyte/macrophages (CD14+) and B lymphocytes (CD19+). Plasmid uptake was verified by temperature-dependent kinetic studies and visualization of internalized plasmid by image-assisted cytometry. Transgene production was detectable only in B lymphocytes, as assessed by qRT-PCR. T lymphocytes co-incubated with B lymphocytes also displayed antigen-specific proliferation and a higher fraction of tetramer-positive CD8 T cells.

## Conclusions

Though plasmid uptake is seen in multiple human cell types, functional antigen production and presentation occurs only in specific cell subsets. These findings suggest that direct antigen presentation upon DNA vaccination might be limited to B lymphocytes. Dendritic cells exhibit robust plasmid DNA uptake, but do not encode the antigen or prime immune responses, potentially playing a detrimental role in immunogenicity. Therefore, these studies highlight several latent points of intervention to improve DNA vaccine induced immunity in humans.

